# *catena*-Poly[zinc-μ_3_-{3,3′-[(1,7-dioxa-4,10-diaza­cyclo­dodecane-4,10-di­yl)bis­(methyl­ene)]dibenzoato}]

**DOI:** 10.1107/S1600536812043450

**Published:** 2012-10-27

**Authors:** C. W. Ingram, L. Liao, J. Bacsa

**Affiliations:** a Center for Functional Nanoscale Materials, Department of Chemistry, Clark Atlanta University, 223 James P. Brawley Drive, Atlanta, GA 30314, USA; b Department of Chemistry, Emory University, Atlanta, GA 30322, USA

## Abstract

The Zn^II^ ion in the title compound, [Zn(C_24_H_28_N_2_O_6_)]_*n*_, is located on a twofold rotation axis and is at the midpoint of a crown-4 moiety of 3,3′-[(1,7-dioxa-4,10-diaza­cyclo­dodecane-4,10-di­yl)bis­(methyl­ene)]dibenzoate anion. It is octahedrally coordinated by two N atoms and two O atoms of the crown moiety from one ligand and two carboxyl­ate O atoms from two bridging intra-chain ligands. Metallomacrocyclic rings are identified in the structure. The metallomacrocycle contains two Zn^II^ ions and 14 atoms from the bridging ligands. Repetition of these units gives rise to an infinite zigzag chain along [101]. C—H⋯O hydrogen bonds occur.

## Related literature

For coordination polymers including metal-organic framework structures, see: Bai *et al.* (2012 ▶); Janiak (2003 ▶); Kitagawa *et al.* (2004 ▶); Li *et al.* (2012 ▶); Liao *et al.* (2012 ▶); Liu *et al.* (2012 ▶); O’Keeffe *et al.* (2000 ▶); Suh *et al.* (2012 ▶); Yoon *et al.* (2012 ▶).
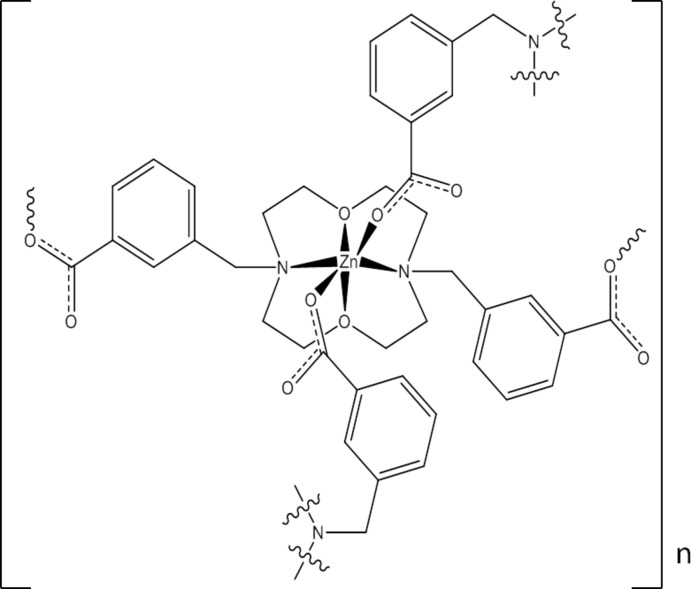


## Experimental

### 

#### Crystal data

[Zn(C_24_H_28_N_2_O_6_)]
*M* *_r_* = 505.87
Monoclinic, 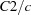

*a* = 20.7264 (15) Å
*b* = 8.9791 (7) Å
*c* = 13.9745 (19) Å
β = 127.200 (4)°
*V* = 2071.5 (4) Å^3^
*Z* = 4
Cu *K*α radiation
μ = 2.05 mm^−1^
*T* = 173 K
0.48 × 0.14 × 0.11 mm

#### Data collection

Bruker D8 diffractometer with an APEXII detector
Absorption correction: multi-scan (*SADABS*; Bruker, 2008 ▶) *T*_min_ = 0.414, *T*_max_ = 0.685
4413 measured reflections
1684 independent reflections
1538 reflections with *I* > 2σ(*I*)
*R*_int_ = 0.029

#### Refinement

*R*[*F* ^2^ > 2σ(*F* ^2^)] = 0.033
*wR*(*F* ^2^) = 0.086
*S* = 1.06
1684 reflections
150 parameters
H-atom parameters constrained
Δρ_max_ = 0.47 e Å^−3^
Δρ_min_ = −0.21 e Å^−3^

### 

Data collection: *APEX2* (Bruker, 2011 ▶); cell refinement: *SAINT* (Bruker, 2009 ▶); data reduction: *SAINT*; program(s) used to solve structure: *SHELXS97* (Sheldrick, 2008 ▶); program(s) used to refine structure: *SHELXL97* (Sheldrick, 2008 ▶); molecular graphics: *OLEX2* (Dolomanov *et al.*, 2009 ▶); software used to prepare material for publication: *OLEX2*.

## Supplementary Material

Crystal structure: contains datablock(s) global, I. DOI: 10.1107/S1600536812043450/mw2087sup1.cif

Structure factors: contains datablock(s) I. DOI: 10.1107/S1600536812043450/mw2087Isup2.hkl

Additional supplementary materials:  crystallographic information; 3D view; checkCIF report

## Figures and Tables

**Table 1:** Hydrogen-bond geometry (Å, °)

*D*—H⋯*A*	*D*—H	H⋯*A*	*D*⋯*A*	*D*—H⋯*A*
C5—H5*A*⋯O1^i^	0.99	2.57	3.224 (3)	124
C12—H12⋯O2^ii^	0.95	2.43	3.256 (3)	145

Symmetry codes: (i) 
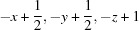
; (ii) 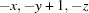.

**Fig. 1.**
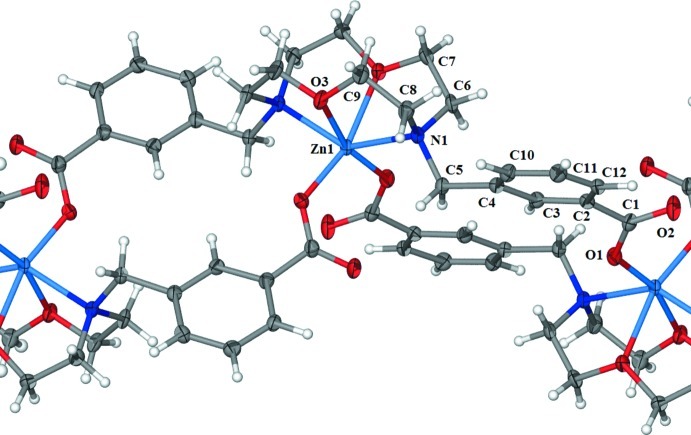
A portion of the one-dimensional chain of 1 showing the 16-membered metallomacrocycle rings.

## supplementary crystallographic information

### Comment

The title compound 1 is the first of a series of coordination polymers that were synthesized from the ligand LH_2_, 3,3'-((1,7-dioxa-4,10-diazacyclododecane-4,10-diyl)bis(methylene)) dibenzoic acid. In these new compounds the metal atoms are positioned in the center of the organic linker.

The asymmetric unit of 1 contains a Zn^II^ ion and a deprotonated ligand L with formula C_24_H_28_N_2_O_6_Zn. The Zn^II^ ion is 6-coordinate in a distorted octahedral geometry being bound to two nitrogen atoms and two oxygen atoms of the crown (1,7-diaza-12-crown-4) and two carboxylic oxygen atoms, one from each of two additional intra-chain ligands (Fig. 1). The Zn1—O1, Zn1—O3 and Zn1—N1 bond lengths are 1.978 (2) Å, 2.287 (2) Å and 2.242 (2) Å, respectively. The O1—Zn1—O1 angle is 107.54 (8)°. The shortest distance between two neighboring Zn^II^ ions in a chain is 9.019 (1) Å.

The Zn^II^ ion of the Zn(crown-4)^2+^ unit is located on a 2-fold rotation axis. The symmetry independent atoms consist of one half of the ligand with the rotation axis generating the second half of the ligand at the Zn atom. Bond circuits consisting of sixteen-membered metallomacrocycle rings can be identified in the structure. Each ring contains two Zn^II^ ions and fourteen non-H atoms of the ligand. Each Zn^II^ ion is a node for three ligands and two connected rings (Fig. 1). The pair of benzene moieties within a metallomacrocycle ring are co-planar within one standard deviation (0.0 (2)°) and the dihedral angle between this plane and the plane of the next two nearest phenyl rings along the 1-D chain is 68.79 (5)°. Repetition of these units creates a 1-D polymer network with an infinite number of these rings.

### Experimental

The title compound was synthesized in an autoclave by mixing the ligand, 3,3'-((1,7-dioxa-4,10-diazacyclododecane-4,10-diyl)bis(methylene))dibenzoic acid, LH_2_ (2x10^-6^ mol), Zn(NO_3_)_2_.6H_2_O (6x10^-6^ mol, 1.79 mg), H_2_O (0.60 ml) and pyridine (2x10^-3^ ml) in a vial of 2 ml capacity. The mixture was heated at 85 °C for 7 d and then cooled to ambient temperature. The white crystals were collected and washed with H_2_O by filtration. Elem. anal. calcd. C_24_H_28_N_2_O_6_Zn %: C, 56.98; H, 5.58; N, 5.54; Found: C, 56.99; H, 5.79; N, 5.58.

### Refinement

Refinement of F^2^ against ALL reflections. The weighted *R*-factor *wR* and goodness of fit S are based on F^2^, conventional *R*-factors *R* are based on F, with F set to zero for negative F^2^. The threshold expression of F^2^ > 2sigma(F^2^) is used only for calculating *R*-factors(gt), *etc* and is not relevant to the choice of reflections for refinement. *R*-factors based on F^2^ are statistically about twice as large as those based on F and *R*- factors based on ALL data will be even larger.

### Crystal data

**Table:**

[Zn(C_24_H_28_N_2_O_6_)]	*F*(000) = 1056
*M**_r_* = 505.87	*D*_x_ = 1.622 Mg m^−^^3^
Monoclinic, *C*2/*c*	Cu *K*α radiation, λ = 1.54184 Å
*a* = 20.7264 (15) Å	Cell parameters from 3719 reflections
*b* = 8.9791 (7) Å	θ = 4.0–67.7°
*c* = 13.9745 (19) Å	µ = 2.05 mm^−^^1^
β = 127.200 (4)°	*T* = 173 K
*V* = 2071.5 (4) Å^3^	Column, colourless
*Z* = 4	0.48 × 0.14 × 0.11 mm

### Data collection

**Table:**

Bruker D8 diffractometer with an APEXII detector	1684 independent reflections
Radiation source: sealed tube	1538 reflections with *I* > 2σ(*I*)
Graphite monochromator	*R*_int_ = 0.029
Detector resolution: 512 pixels mm^-1^	θ_max_ = 65.1°, θ_min_ = 5.4°
φ and ω scans with a narrow frame width	*h* = −24→18
Absorption correction: multi-scan (*SADABS*; Bruker, 2008)	*k* = −10→10
*T*_min_ = 0.414, *T*_max_ = 0.685	*l* = −14→15
4413 measured reflections	

### Refinement

**Table:**

Refinement on *F*^2^	Primary atom site location: structure-invariant direct methods
Least-squares matrix: full	Secondary atom site location: difference Fourier map
*R*[*F*^2^ > 2σ(*F*^2^)] = 0.033	Hydrogen site location: inferred from neighbouring sites
*wR*(*F*^2^) = 0.086	H-atom parameters constrained
*S* = 1.06	*w* = 1/[σ^2^(*F*_o_^2^) + (0.0475*P*)^2^ + 2.6887*P*] where *P* = (*F*_o_^2^ + 2*F*_c_^2^)/3
1684 reflections	(Δ/σ)_max_ < 0.001
150 parameters	Δρ_max_ = 0.47 e Å^−^^3^
0 restraints	Δρ_min_ = −0.21 e Å^−^^3^

### Special details

**Table:**

Experimental. Absorption correction: SADABS (Bruker-AXS, 2008) was used for absorption correction. R(int) was 0.0732 before and 0.0388 after correction. The ratio of minimum to maximum transmission is 0.6049. The λ/2 correction factor is not present.
Geometry. Bond distances, angles etc. have been calculated using the rounded fractional coordinates. All su's are estimated from the variances of the (full) variance-covariance matrix. The cell esds are taken into account in the estimation of distances, angles and torsion angles
Refinement. Refinement of *F*^2^ against ALL reflections. The weighted *R*-factor *wR* and goodness of fit *S* are based on *F*^2^, conventional *R*-factors *R* are based on *F*, with *F* set to zero for negative *F*^2^. The threshold expression of *F*^2^> σ(*F*^2^) is used only for calculating *R*-factors(gt) *etc*. and is not relevant to the choice of reflections for refinement. *R*-factors based on *F*^2^ are statistically about twice as large as those based on *F*, and *R*- factors based on ALL data will be even larger.

### Fractional atomic coordinates and isotropic or equivalent isotropic displacement parameters (Å^2^)

**Table:**

	*x*	*y*	*z*	*U*_iso_*/*U*_eq_	
Zn1	0.00000	0.05167 (4)	0.25000	0.0179 (1)	
O1	0.07654 (9)	0.18185 (17)	0.24895 (14)	0.0224 (4)	
O2	−0.00673 (9)	0.3592 (2)	0.11945 (15)	0.0325 (5)	
O3	0.55621 (9)	0.63747 (19)	0.71322 (13)	0.0254 (4)	
N1	0.39893 (11)	0.5343 (2)	0.56752 (16)	0.0193 (5)	
C1	0.06066 (13)	0.3070 (3)	0.19486 (19)	0.0213 (6)	
C2	0.13504 (13)	0.3887 (2)	0.22567 (19)	0.0196 (6)	
C3	0.21035 (13)	0.3696 (2)	0.33678 (19)	0.0201 (6)	
C4	0.28068 (13)	0.4328 (2)	0.36223 (19)	0.0200 (6)	
C5	0.36142 (13)	0.4041 (2)	0.48377 (19)	0.0206 (6)	
C6	0.33847 (13)	0.5969 (3)	0.58104 (19)	0.0232 (6)	
C7	0.37416 (14)	0.7072 (3)	0.6834 (2)	0.0258 (7)	
C8	0.42804 (14)	0.6450 (3)	0.52237 (19)	0.0247 (6)	
C9	0.49985 (14)	0.7382 (3)	0.6195 (2)	0.0255 (7)	
C10	0.27460 (13)	0.5139 (3)	0.2717 (2)	0.0229 (6)	
C11	0.19960 (14)	0.5358 (3)	0.1619 (2)	0.0235 (7)	
C12	0.12995 (13)	0.4758 (2)	0.13890 (19)	0.0204 (6)	
H3	0.21390	0.31190	0.39680	0.0240*	
H5A	0.35370	0.32330	0.52420	0.0250*	
H5B	0.40020	0.36750	0.46990	0.0250*	
H6A	0.31330	0.51420	0.59450	0.0280*	
H6B	0.29520	0.64700	0.50520	0.0280*	
H7A	0.38980	0.80030	0.66420	0.0310*	
H7B	0.33420	0.73180	0.69810	0.0310*	
H8A	0.38270	0.71270	0.46570	0.0300*	
H8B	0.44340	0.59150	0.47690	0.0300*	
H9A	0.52510	0.78940	0.58690	0.0310*	
H9B	0.48220	0.81430	0.65050	0.0310*	
H10	0.32190	0.55410	0.28530	0.0280*	
H11	0.19600	0.59300	0.10160	0.0280*	
H12	0.07880	0.49400	0.06410	0.0250*	

### Atomic displacement parameters (Å^2^)

**Table:**

	*U*^11^	*U*^22^	*U*^33^	*U*^12^	*U*^13^	*U*^23^
Zn1	0.0119 (2)	0.0195 (2)	0.0178 (2)	0.0000	0.0066 (2)	0.0000
O1	0.0161 (7)	0.0227 (8)	0.0279 (8)	−0.0020 (6)	0.0131 (7)	0.0024 (6)
O2	0.0159 (8)	0.0391 (10)	0.0287 (9)	−0.0006 (7)	0.0063 (7)	0.0110 (7)
O3	0.0178 (8)	0.0244 (8)	0.0205 (7)	−0.0034 (6)	0.0045 (7)	0.0021 (6)
N1	0.0142 (9)	0.0212 (10)	0.0184 (9)	0.0003 (7)	0.0077 (8)	0.0012 (7)
C1	0.0181 (11)	0.0250 (12)	0.0170 (10)	−0.0017 (9)	0.0086 (9)	−0.0012 (9)
C2	0.0181 (11)	0.0191 (11)	0.0197 (10)	0.0019 (8)	0.0104 (9)	−0.0012 (8)
C3	0.0198 (11)	0.0198 (11)	0.0183 (10)	0.0003 (9)	0.0103 (9)	0.0006 (8)
C4	0.0174 (11)	0.0195 (11)	0.0197 (10)	0.0005 (8)	0.0094 (9)	−0.0016 (8)
C5	0.0154 (10)	0.0218 (11)	0.0192 (10)	0.0000 (8)	0.0077 (9)	0.0007 (8)
C6	0.0129 (10)	0.0291 (12)	0.0198 (10)	0.0051 (9)	0.0058 (9)	0.0020 (9)
C7	0.0181 (11)	0.0279 (12)	0.0226 (11)	0.0082 (9)	0.0077 (9)	0.0021 (9)
C8	0.0207 (11)	0.0264 (12)	0.0190 (10)	−0.0014 (9)	0.0078 (10)	0.0043 (9)
C9	0.0217 (11)	0.0237 (12)	0.0234 (11)	−0.0022 (9)	0.0096 (10)	0.0059 (9)
C10	0.0198 (11)	0.0249 (11)	0.0233 (11)	−0.0033 (9)	0.0126 (10)	−0.0020 (9)
C11	0.0244 (12)	0.0239 (12)	0.0208 (11)	−0.0004 (9)	0.0130 (10)	0.0022 (9)
C12	0.0170 (11)	0.0197 (11)	0.0180 (10)	0.0021 (8)	0.0071 (9)	−0.0008 (8)

### Geometric parameters (Å, º)

**Table:**

Zn1—O1	1.978 (2)	C6—C7	1.515 (3)
Zn1—O1^i^	1.978 (2)	C8—C9	1.522 (4)
Zn1—O3^ii^	2.2869 (19)	C10—C11	1.388 (4)
Zn1—N1^ii^	2.2422 (19)	C11—C12	1.384 (4)
Zn1—O3^iii^	2.2869 (19)	C3—H3	0.9500
Zn1—N1^iii^	2.2422 (19)	C5—H5A	0.9900
O1—C1	1.281 (3)	C5—H5B	0.9900
O2—C1	1.225 (3)	C6—H6A	0.9900
O3—C9	1.431 (3)	C6—H6B	0.9900
O3—C7^iv^	1.429 (3)	C7—H7A	0.9900
N1—C5	1.497 (3)	C7—H7B	0.9900
N1—C6	1.486 (4)	C8—H8A	0.9900
N1—C8	1.487 (4)	C8—H8B	0.9900
C1—C2	1.516 (4)	C9—H9A	0.9900
C2—C3	1.395 (3)	C9—H9B	0.9900
C2—C12	1.392 (3)	C10—H10	0.9500
C3—C4	1.395 (4)	C11—H11	0.9500
C4—C5	1.521 (3)	C12—H12	0.9500
C4—C10	1.398 (3)		
			
O1—Zn1—O1^i^	107.54 (8)	N1—C8—C9	114.72 (19)
O1—Zn1—O3^ii^	163.85 (7)	O3—C9—C8	106.6 (2)
O1—Zn1—N1^ii^	90.57 (8)	C4—C10—C11	120.2 (3)
O1—Zn1—O3^iii^	85.26 (7)	C10—C11—C12	121.0 (2)
O1—Zn1—N1^iii^	113.40 (8)	C2—C12—C11	119.8 (2)
O1^i^—Zn1—O3^ii^	85.26 (7)	C2—C3—H3	119.00
O1^i^—Zn1—N1^ii^	113.40 (8)	C4—C3—H3	119.00
O1^i^—Zn1—O3^iii^	163.85 (7)	N1—C5—H5A	108.00
O1^i^—Zn1—N1^iii^	90.57 (8)	N1—C5—H5B	108.00
O3^ii^—Zn1—N1^ii^	75.01 (7)	C4—C5—H5A	108.00
O3^ii^—Zn1—O3^iii^	84.09 (7)	C4—C5—H5B	108.00
O3^ii^—Zn1—N1^iii^	75.37 (7)	H5A—C5—H5B	107.00
O3^iii^—Zn1—N1^ii^	75.37 (7)	N1—C6—H6A	109.00
N1^ii^—Zn1—N1^iii^	139.73 (7)	N1—C6—H6B	109.00
O3^iii^—Zn1—N1^iii^	75.01 (7)	C7—C6—H6A	109.00
Zn1—O1—C1	126.99 (19)	C7—C6—H6B	109.00
C7^iv^—O3—C9	114.52 (19)	H6A—C6—H6B	108.00
Zn1^ii^—O3—C9	115.48 (17)	C6—C7—H7A	110.00
Zn1^ii^—O3—C7^iv^	116.02 (14)	C6—C7—H7B	110.00
C5—N1—C6	108.4 (2)	H7A—C7—H7B	109.00
C5—N1—C8	110.03 (19)	O3^iv^—C7—H7A	110.00
Zn1^ii^—N1—C5	107.87 (12)	O3^iv^—C7—H7B	110.00
C6—N1—C8	113.0 (2)	N1—C8—H8A	109.00
Zn1^ii^—N1—C6	105.39 (13)	N1—C8—H8B	109.00
Zn1^ii^—N1—C8	111.90 (16)	C9—C8—H8A	109.00
O1—C1—O2	126.6 (3)	C9—C8—H8B	109.00
O1—C1—C2	113.7 (2)	H8A—C8—H8B	108.00
O2—C1—C2	119.6 (2)	O3—C9—H9A	110.00
C1—C2—C3	121.2 (2)	O3—C9—H9B	110.00
C1—C2—C12	119.7 (2)	C8—C9—H9A	110.00
C3—C2—C12	118.9 (3)	C8—C9—H9B	110.00
C2—C3—C4	121.8 (2)	H9A—C9—H9B	109.00
C3—C4—C5	119.4 (2)	C4—C10—H10	120.00
C3—C4—C10	118.2 (2)	C11—C10—H10	120.00
C5—C4—C10	122.4 (3)	C10—C11—H11	119.00
N1—C5—C4	116.19 (17)	C12—C11—H11	120.00
N1—C6—C7	113.6 (2)	C2—C12—H12	120.00
O3^iv^—C7—C6	106.6 (2)	C11—C12—H12	120.00
			
O1^i^—Zn1—O1—C1	−36.0 (2)	O1—C1—C2—C3	−28.4 (3)
N1^ii^—Zn1—O1—C1	−150.78 (19)	O1—C1—C2—C12	146.7 (2)
O3^iii^—Zn1—O1—C1	133.96 (19)	O2—C1—C2—C3	155.2 (2)
N1^iii^—Zn1—O1—C1	62.5 (2)	O2—C1—C2—C12	−29.7 (4)
Zn1—O1—C1—O2	−11.7 (4)	C1—C2—C3—C4	173.8 (2)
Zn1—O1—C1—C2	172.18 (15)	C12—C2—C3—C4	−1.3 (3)
C7^iv^—O3—C9—C8	−176.1 (2)	C1—C2—C12—C11	−172.2 (2)
Zn1^ii^—O3—C9—C8	−37.4 (3)	C3—C2—C12—C11	3.0 (3)
C9—O3—C7^iv^—C6^iv^	160.4 (2)	C2—C3—C4—C5	−178.2 (2)
C6—N1—C5—C4	53.7 (3)	C2—C3—C4—C10	−1.7 (3)
C8—N1—C5—C4	−70.4 (3)	C3—C4—C5—N1	−110.1 (2)
Zn1^ii^—N1—C5—C4	167.3 (2)	C10—C4—C5—N1	73.5 (3)
C5—N1—C6—C7	167.45 (19)	C3—C4—C10—C11	3.0 (4)
C8—N1—C6—C7	−70.3 (2)	C5—C4—C10—C11	179.4 (2)
Zn1^ii^—N1—C6—C7	52.2 (2)	N1—C6—C7—O3^iv^	−49.9 (3)
C5—N1—C8—C9	−151.4 (2)	N1—C8—C9—O3	45.1 (3)
C6—N1—C8—C9	87.3 (3)	C4—C10—C11—C12	−1.3 (4)
Zn1^ii^—N1—C8—C9	−31.5 (3)	C10—C11—C12—C2	−1.7 (4)

Symmetry codes: (i) −*x*, *y*, −*z*+1/2; (ii) −*x*+1/2, −*y*+1/2, −*z*+1; (iii) *x*−1/2, −*y*+1/2, *z*−1/2; (iv) −*x*+1, *y*, −*z*+3/2.

### Hydrogen-bond geometry (Å, º)

**Table:**

*D*—H···*A*	*D*—H	H···*A*	*D*···*A*	*D*—H···*A*
C5—H5*A*···O1^ii^	0.99	2.57	3.224 (3)	124
C12—H12···O2^v^	0.95	2.43	3.256 (3)	145

Symmetry codes: (ii) −*x*+1/2, −*y*+1/2, −*z*+1; (v) −*x*, −*y*+1, −*z*.
